# Variable Phenotypes of Epilepsy, Intellectual Disability, and Schizophrenia Caused by 12p13.33–p13.32 Terminal Microdeletion in a Korean Family: A Case Report and Literature Review

**DOI:** 10.3390/genes12071001

**Published:** 2021-06-29

**Authors:** Ji Yoon Han, Joonhong Park

**Affiliations:** 1 Department of Pediatrics, College of Medicine, The Catholic University of Korea, Seoul 06591, Korea; han024@catholic.ac.kr; 2 Department of Laboratory Medicine, Jeonbuk National University Medical School and Hospital, Jeonju 54907, Korea; 3 Research Institute of Clinical Medicine of Jeonbuk National University-Biomedical Research Institute of Jeonbuk National University Hospital, Jeonju 54907, Korea

**Keywords:** variable phenotypes, 12p13.33 microdeletion, epilepsy, intellectual disability, schizophrenia, exome sequencing, chromosomal microarray, *CACNA1C* gene, *KDM5A* gene

## Abstract

A simultaneous analysis of nucleotide changes and copy number variations (CNVs) based on exome sequencing data was demonstrated as a potential new first-tier diagnosis strategy for rare neuropsychiatric disorders. In this report, using depth-of-coverage analysis from exome sequencing data, we described variable phenotypes of epilepsy, intellectual disability (ID), and schizophrenia caused by 12p13.33–p13.32 terminal microdeletion in a Korean family. We hypothesized that *CACNA1C* and *KDM5A* genes of the six candidate genes located in this region were the best candidates for explaining epilepsy, ID, and schizophrenia and may be responsible for clinical features reported in cases with monosomy of the 12p13.33 subtelomeric region. On the background of microdeletion syndrome, which was described in clinical cases with mild, moderate, and severe neurodevelopmental manifestations as well as impairments, the clinician may determine whether the patient will end up with a more severe or milder end-phenotype, which in turn determines disease prognosis. In our case, the 12p13.33–p13.32 terminal microdeletion may explain the variable expressivity in the same family. However, further comprehensive studies with larger cohorts focusing on careful phenotyping across the lifespan are required to clearly elucidate the possible contribution of genetic modifiers and the environmental influence on the expressivity of 12p13.33 microdeletion and associated characteristics.

## 1. Introduction

Developmental delay (DD) and intellectual disability (ID) are key features in patients with subtelomeric defects. Subtelomeric regions are more susceptible to aberrant alterations than other chromosomal regions and are commonly enriched for genes [1]. For example, 4p- (Wolf-Hirschhorn), 5p- (cri du chat), 9p-, 13q-, and 18p- syndromes are cases of microscopically visible deletions in the subtelomeric region, which usually cause DD or ID [2]. Although the disease and genetic association have not been well defined, subtelomeric alterations were reported as a cause of DD, ID, and multiple congenital anomalies [3,4]. In these cases, the clinical manifestations are probably determined by the type and location of the chromosomal alterations, such as duplications or deletions, as well as the size of the alterations, including the function and numbers of the genes involved [5]. Fine mapping of the subtelomeric regions has become a new strategy for identifying novel genes responsible for DD and/or ID. Constitutional deletions involving the distal part of the short arm of chromosome 12 (12p13.33–p13.32) are very rare and have been reported in only 28 cases so far [6,7,8,9,10,11,12,13,14,15,16,17,18,19,20,21,22,23]; either inherited [14,15,17] or sporadic [12,16,17,21,23] and identified by conventional karyotyping or chromosomal microarray (CMA). Notably, among the reported cases, the clinical manifestations differed substantially according to the size of the deletion. In addition, a distinct number of similar phenotypes were outlined, resulting in the proposal of a 12p13 deletion spectrum syndrome [13,21], which are generally characterized by a variable degree of behavioral problems and delayed speech or language development with or without ID. Nevertheless, several confounders in drafting a list of frequent clinical symptoms were illustrated, based on the failure to account for the size of deletions sizes and missing differences between 12p13 terminal and interstitial microdeletions. Differential haploinsufficiency of a bunch of these genes acting together is responsible for the variable phenotypes of cases with monosomy of the 12p13.33 subtelomeric region. In the last decades, comparisons of sporadic 12p13.33 microdeletions proposed a novel 12p13.33 locus that is associated with childhood speech apraxia and harbors nine genes, including *ADIPOR2*, *CACNA1C*, *CACNA2D4*, *DCP1B*, *ERC1*, *FBXL14*, *LRTM2*, *RPS4X*, and *WNT5B* [12,14,17,18,22]. 

On the other hand, next-generation sequencing (NGS), including the low-coverage whole-genome sequencing (WGS) approach, is more sensitive to detecting copy number variations (CNVs) as compared with the currently commercially available chromosomal microarrays and is effective for CNV detection in clinical samples [24]. Furthermore, simultaneous analysis of nucleotide changes and CNVs based on exome sequencing data was demonstrated as a potential new first-tier strategy to diagnose rare neuropsychiatric disorders [25]. The combination of copy number variation sequencing and exome sequencing is recommended as an improved diagnostic option for cases with less obvious or otherwise undiagnosed diseases suspected to have a genetic etiology [26].

In this report, using depth-of-coverage analysis from exome sequencing data, we identified a putative 12p13.33–p13.32 terminal microdeletion in a Korean family with epilepsy, ID, and schizophrenia. We hypothesized that exome sequencing analysis and comparative genetic evaluation of reported cases with 12p13.33–p13.32 microdeletion would provide sufficient data to confirm that *CACNA1C* and *KDM5A* genes of the six candidate genes located in this region were the best candidates for explaining epilepsy, ID, and schizophrenia and may be responsible for clinical features reported in cases with monosomy of the 12p13.33 subtelomeric region.

## 2. Materials and Methods

### 2.1. Exome Sequencing of the Mother–Daughter Duo

To investigate the potential genetic cause of epilepsy, ID and/or schizophrenia in this family, the exomic DNA of the proband and her mother were enriched using Agilent’s SureSelect XT Human All Exon v5 (Agilent Technologies, Santa Clara, CA, USA). Based on neuropsychiatric disorder suspicions, paired-end sequencing was conducted on the Illumina HiSeq2500 (Illumina, San Diego, CA, USA) at the Green Cross Genome (Yongin, Korea) to identify the genetic alteration. Base-calling, alignment, variant calling, annotation, and quality control reporting were performed using a GATK Best Practices workflow for germline short variant discovery (https://gatk.broadinstitute.org/hc/en-us; accessed on 27 August 2020). Interpretation of sequence variants was manually reviewed by medical laboratory geneticists according to the Joint Consensus Recommendation of the American College of Medical Genetics and Genomics and the Association for Molecular Pathology standards and guidelines [27]. The filtering criteria for nucleotide changes were: Phred quality score >20, no Fisher strand bias, read depth >30×, allele frequency <0.1%, non-synonymous substitution or indel occurred in coding region and exon–intron boundaries, heterozygous variant in both the mother and proband in a dominant manner, and reported to be associated with epilepsy, DD/ID, and/or schizophrenia in the OMIM database. Each of the candidate variants leading to a genetic diagnosis was visually evaluated using Integrative Genomics Viewer followed by Sanger validation. Candidate variant genes with predicted pathogenicity and those affecting the coding region or splice site were validated using Sanger sequencing with ABI3500XL Genetic Analyzer (Applied Biosystems, Foster City, CA, USA). Considering that unexplained DD/ID, congenital anomalies, and autism spectrum disorder (ASD) have been associated with CNV [28], an additional depth-of-coverage analysis of exome sequencing data was performed using ExomeDepth, call CNVs from targeted sequence data [29], and VisCap, inference, and visualization of germline CNVs from targeted clinical sequencing data [30].

### 2.2. Chromosomal Microarray

To confirm the CNV identified by exome sequencing with depth-of-coverage analysis, a whole genomic screening of chromosomal rearrangements by CMA was performed using SurePrint G3 Human CGH + SNP Microarray 4 × 180K (Agilent Technologies, Santa Clara, CA, USA), according to the manufacturers’ recommendation. All the samples were matched with Human Genomic DNA reference (Agilent Technologies or Promega, Madison, WI, USA). Data were obtained using the Agilent Feature Extraction software 12.0.2.2 and Agilent CytoGenomics 4.0 and visually assessed using the Agilent Genomic Workbench Software 7.0.4.0 and Agilent CytoGenomics 4.0. Genomic positions were mapped using the human genomic reference sequence GRCh37/hg19. CNVs were identified using the ADM-2 algorithm with filters of minimal absolute average log ratio of 0.25 as the cut-off, copy number neutral loss of heterozygosity regions, and minimal size of 200 kb in the region.

## 3. Case Presentation

**Figure 1:**
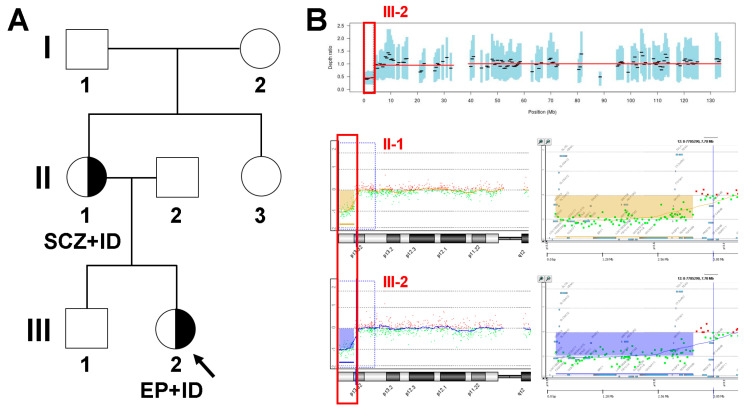
Pedigree analysis and coverage analysis using exome sequencing data and array–comparative genomic hybridization in the proband (III-2) and her mother (II-1). (**A**) Family pedigree shows a putative 12p13.33–p13.32 terminal microdeletion in a dominant manner in Korean families with epilepsy, intellectual disability, and schizophrenia. SCZ, schizophrenia; ID, intellectual disability; EP, epilepsy. (**B**) Results of copy number analysis using exome sequencing data and chromosomal microarray. (Upper panel) Copy number analysis using exome sequencing data detected 12p13.33–p13.32 terminal microdeletion in the proband, as highlighted in the red box. (Lower panel) Additional chromosomal microarray identified 12p13.33–p13.32 terminal microdeletion in the proband and her mother. The arr[hg19]12p13.33p13.32(230,421_3,394,129)x1 encompasses 26 genes, including *IQSEC3*, *SLC6A12*, *SLC6A13*, *KDM5A*, *CCDC77*, *B4GALNT3*, *NINJ2*, *WNK1*, *RAD52*, *ERC1*, *WNT5B*, *FBXL14*, *ADIPOR2*, *CACNA2D4*, *LRTM2*, *DCP1B*, *CACNA1C*, *FKBP4*, *ITFG2*, *NRIP2*, *TEX52*, *FOXM1*, *RHNO1*, *TULP3*, *TEAD4*, and *TSPAN9*, as highlighted in the red box.

The proband (III-2 in Figure 1A) was a 12-year-old girl with status epilepticus and ID, who was referred to the department of pediatric neurology, Daejeon St. Mary’s Hospital (Daejeon, Korea), due to prolonged seizures. She showed generalized tonic-clonic seizure with upper eyeball deviation for 30 minutes. Seizure ceased after administration of lorazepam, phenytoin, and phenobarbital. She was the second child of non-consanguineous parents, and pregnancy was uneventful. Except for the mother’s history of neuropsychiatric disorder, there was no family history of neurodevelopmental diseases, including epilepsy, DD/ID, or psychiatric disorder. She had neither facial dysmorphism nor skeletal malformations on the physical examination. There was no problem during the neonatal period: She smiled at 12 weeks, sat at 10 months, crawled at 17 months, and walked independently at 24 months. Language development was profoundly delayed with first words at 3 years. She was not fully toilet trained until the age of 9 years. Standard education was interrupted before the child started primary school, and she attended a school for special-needs children. Her intellectual quotient was estimated at 60, indicating moderate ID on the Wechsler Intelligence Scale for Children—Revised. Brain magnetic resonance imaging showed a normal myelination pattern with no obvious abnormalities. Electroencephalography revealed sharp-and-slow waves on the left central regions. Visual and auditory evoked potentials were normal. Electrocardiography and echocardiogram showed normal results. Seizures were controlled phenobarbital and levetiracetam. On the other hand, the 40-year-old proband’s mother (II-1 in Figure 1A) had mild ID and schizophrenia. She was the first child of healthy, non-consanguineous parents and was born at 38 weeks via Caesarean section. Her younger sister was healthy and had normal intelligence. Her physical examination results were normal without any dysmorphic features or malformation. She had difficulties schooling but did not attend a special-needs school. She was not able to achieve a high school curriculum but followed a vocational course. She worked as a clerk in a grocery store and married at 24 years old. When she was about 25 years old, she showed confused thinking, visual and auditory hallucinations. An assessment at 26 years of age confirmed schizophrenia, and she has been taking risperidone and fluoxetine. Now she does not work and lives as a housewife. She did not experience seizures during her adolescence years and early adulthood. Careful laboratory and radiology etiologic investigations were performed on the proband and her mother, and all the results were within the normal range. In addition, the chromosomal analysis revealed normal female karyotype, Fragile X testing was negative, and metabolic laboratory test results were within the normal limits.

## 4. Results

On-target yields of 3,415,455,900 and 3,386,597,831 reads were generated from the proband and her mother by estimating the sequence quality along all sequences. The mean read depths (×) were 68 and 65, and the percentage of bases above 30× was 84% and 82%, respectively. The duo exome sequencing identified no likely pathogenic variant associated with epilepsy, ID, and schizophrenia as a dominant inheritance. However, both of the CNV detection tools identified a 3.2 Mb heterozygous deletion at chromosome 12p13.33–p13.32 with a Z ratio between −0.5 and −1. As a complementary alternative method, CMA confirmed a terminal microdeletion at 12p13.33–p13.32 described as arr[hg19]12p13.33p13.32(230,421_3,394,129)x1 in the proband and her mother. This 12p13.33–p13.32 terminal microdeletion in a Korean family encompassed 26 genes, including *IQSEC3*, *SLC6A12*, *SLC6A13*, *KDM5A*, *CCDC77*, *B4GALNT3*, *NINJ2*, *WNK1*, *RAD52*, *ERC1*, *WNT5B*, *FBXL14*, *ADIPOR2*, *CACNA2D4*, *LRTM2*, *DCP1B*, *CACNA1C*, *FKBP4*, *ITFG2*, *NRIP2*, *TEX52*, *FOXM1*, *RHNO1*, *TULP3*, *TEAD4*, and *TSPAN9*. (Figure 1B).

**Figure 2:**
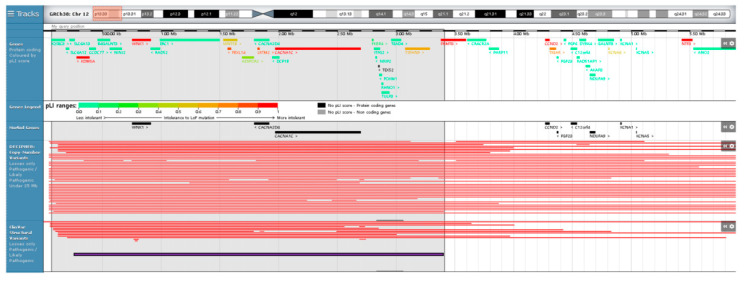
Schematic representation of our patient’s deletion compared with the previously reported cases spanning the terminal 5.5 Mb of chromosome 12p displayed by DECIPHER’s genome browser (https://decipher.sanger.ac.uk/browser#q/12:66711-3409277/location/12:1-7952122; accessed on 11 March 2021). Red bars indicate pathogenic/like pathogenic losses under 25 Mb by Decipher and ClinVar. Violet bar indicates 12p13.33–p13.32 identified in this study and involves six candidate genes with pLI score >0.9 and/or gene morbidities such as *CACNA1C*, *KDM5A*, *WNK1*, *FBXL14*, *LRTM2*, and *CACNA2D4*.

Among 26 genes located on 12p13.33–p13.32 terminal microdeletion in a Korean family, candidate genes expected to be related to epilepsy, ID, and schizophrenia were selected according to the probability of being loss-of-function intolerant (pLI) score and gene morbidity of corresponding genes provided by DECIPHER’s genome browser (https://decipher.sanger.ac.uk/browser#q/12:66711-3409277/location/12:1-7952122; accessed on 11 March 2021) [31]. As a result, six candidate genes with pLI score >0.9 and/or gene morbidity were proposed to be associated with the clinical phenotypes in a Korean family: *CACNA1C*, *KDM5A*, *WNK1*, *FBXL14*, *LRTM2*, and *CACNA2D4*. Schematic representation of our patients’ microdeletion, compared with previously reported cases spanning the terminal 5.5 Mb of chromosome 12p13, was displayed by DECIPHER’s genome browser (Figure 2).

## 5. Discussion

Cytogenetically chromosomal deletions involving the distal region of chromosome 12p13 are relatively uncommon [15]. The first case was published in 1974 with clinical manifestations including ID, atrial septal defect, microcephaly, and facial dysmorphism [6]. To date, approximately 28 reported cases with 12p13.33 microdeletion have been described and are associated with DD/ID and various neuropsychiatric manifestations (Table 1). In these cases [6,7,8,9,10,11,12,13,14,15,16,17,18,19,20,21,22,23], the phenotypic features were variable and indistinct, maybe due to the size of the deleted segment. However, there seems to be no correlation between the size of the deleted size and the severity of the reported clinical phenotypes. Within-family discordant expressivity, age-dependent presentation, and combined functional effects of multiple haploinsufficient genes can be attributed to the reported phenotypic variability [15,17,22]. Nearly 60% of cases with 12p13.33 microdeletion had facial dysmorphism such as low-set ears, micrognathia, and slant of palpebral fissures. In the present study, approximately 30% behavioral problems incidence rate was reported in cases with 12p13.33 microdeletion, while schizophrenia accounted for 10% of the cases. No definite malformations were noted in non-specific dysmorphic features. Our patients showed variable clinical phenotype and disease severity, which might be partly related to a post-conceptual somatic mutation, an epigenetic change, an environmental factor, or as yet defined genetic abnormalities. Seizures were not previously described elsewhere apart from when our proband had epilepsy and was managed with two anti-seizure medications.

The 12p13 subtelomeric region is not a gene-abundant region and has about 35 genes spanning the four telomeric megabases. Of the six candidate genes located in this region, *CACNA1C* and *KDM5A* genes were the best candidates for explaining epilepsy, ID, and schizophrenia in a Korean family. The *CACNA1C* gene encodes the alpha-1 subunit of L-type voltage-dependent calcium channel (as known as Cav1.2) that is widely expressed in the brain (predominantly in the hippocampus, thalamus, and cerebral cortex) [32]. The Cav1.2 channel plays a part in neurotransmitter release, synaptogenesis, and neuronal excitability; therefore, mutation of *CACNA1C* is a possible cause of neuropsychiatric disorders [33]. Recently, the *CACNA1C* haploinsufficiency was reported to be responsible for the common features of interstitial 12p13.33 deletion carriers [23]. Furthermore, rs1006737 or the *CACNA1C* and rs1344706 ZNF804A were commonly associated with schizophrenia and bipolar disorder, and recently with brain phenotypes. For instance, *CACNA1C* up-regulation in schizophrenia and bipolar disorder parietal cortices [33] and implication of *CACN1C* variants in Timothy syndrome, depression, schizophrenia, bipolar disorder, and impairment of working memory and verbal fluency [34,35]. The *CACN1C* has potential pathogenicity in corresponding epilepsy [36]; two families with *CACNA1C* mutation were presented with neonatal epileptic encephalopathy and/or epilepsy combined DD/ID [37]. The *CACNA1C* could in part explain clinical manifestations in our proband [38], therefore we propose *CACN1C* as a new candidate gene contributing to epilepsy, ID, and schizophrenia. A detailed molecular analysis of *CACN1C* transcripts is required to establish its functional role in the neurotransmission process and its etiological association with neuropsychiatric disabilities. 

On the other hand, mutations in several genes coding for epigenetic modulators and chromatin remodelers were associated with neurodevelopmental diseases such as ASD [39,40]. Among them, the *KDM5A* gene encodes a chromatin regulator, which belongs to the KDM5 family of lysine-specific histone H3 demethylases. Histone H3 lysine 4 trimethyl (H3K4me3) marks are present at active enhancers and gene promoters and are regulated by multiple factors such as the KDM5 family members *KDM5B* and *KDM5C* [41]. Mutations in lysine demethylases regulating the demethylation of the H3K4me3 marks were associated strongly with a host of neurodevelopmental diseases. Two genes of the *KDM5B* and *KDM5C* in the KDM5 family are disrupted in neurodevelopmental diseases such as ASD and ID [42,43,44]. Thus, mutations in *KDM5A*, *KDM5B*, and *KDM5C* lead to neurodevelopmental diseases, indicates that their function in the brain is essential. Particularly, KDM5A demethylates H3K4me3, a histone mark known to be associated with memory retrieval [45] and memory formation [46]. Several pathogenic *KDM5A* mutations were reported previously in patients with ASD, in conjunction with lack of speech, DD, and/or ID. Despite the phenotypic diversity observed commonly within the different genetic subtypes of ASD, all the patients with *KDM5A* mutations reported in a recent study show a complete absence of speech, suggesting a critical role for KDM5A in regulating verbal communication [47]. Perturbations in histone demethylases and methyltransferases at this mark give rise to neurodevelopmental diseases, including ID and ASD [48], emphasizing the significance of H3K4me3 in memory and learning. 

Interestingly, CNV analysis based on the NGS approach could show several unrelated phenotypes together simply as the result of the probabilistic occurrence. Disease phenotypes mainly led to a single driver alteration, which mostly determines the diagnosis, but amelioration or exacerbation by epistatic effects is expected. On the background of microdeletion syndrome, which was described in clinical cases with mild, moderate, and severe neurodevelopmental manifestations as well as impairments, the clinician may determine whether the patient will end up with a more severe or milder end-phenotype that variably determine disease prognosis [49]. In our case, the 12p13.33–p13.32 terminal microdeletion may explain the variable expressivity in the same family. The physician should be aware that seizures and neuropsychiatric problems may occur with age in children with 12p13.33–p13.32 deletion. A better understanding of the functional consequence of 12p13.33–p13.32 deletion is needed for the targeted medicinal and non-medicinal therapy.

## 6. Conclusions

In the present study, we identified variable phenotypes of epilepsy, ID, and schizophrenia caused by 12p13.33–p13.32 terminal microdeletion in a Korean family. Based on our findings, we propose that the haploinsufficiency of protein-coding genes might have a cardinal role in all the common characteristic phenotypes, such as expressive language delay, because these genes are have been reported to be entirely deleted in cases of 12p13.33 microdeletions or located in diverse deletion breakpoint loci. However, further comprehensive studies with larger cohorts focusing on careful phenotyping across the lifespan are required to clearly elucidate the possible contribution of genetic modifiers and the environmental influence on the expressivity of 12p13.33 microdeletion and associated characteristics.

## Figures and Tables

**Table 1:** Literature review of clinical manifestations in cases with 12p.13.3 microdeletions.

Literatures	Age (yr)	Sex	Parental Age (M/F)	Methods	Segment	Size	Clinical Manifestations	Remarks
Mayeda (1974) [6]	10	F	27/28	Q-banding	del(12)p13pter	-	Strabismus, hypoplastic mandible, large low-set ear, crowed mal-aligned teeth, a funnel chest, atrial septal defect, hypoplastic right lung, short metacarpal bone	Recurrent infections
Magnelli (1975) [7]	35	M	34/41	Q-banding	del(12)p13pter	-	Short stature, microcephaly, antimongoloid slant of palpebral fissures, imperfect dental enamel, short and webbed neck, short arm and short hands, brachymetaphalangy, short 2nd fingers, broad thumb, short metatarsal bones, big first toes	
Kivlin (1985) [8]	3	F	22/-	Q-banding	del(12)(qter→p12.2)	-	ID, microcephaly, micrognathia, sclerocornea, omphalocele, bilateral choanal atresia, simian lines, clinodactyly of toes	
Romain (1987) [9]	3	F	26/26	G-banding	del(12)p13.1-p13.3	-	DD, protruding tongue, strabismus, large mouth, unusual tooth, slightly micrognathia	
Baker (2002) [10]	15	M	-/-	G-banding, FISH	del(12)p13.33	1.65 Mb	ID, deep-set ear, prominent ears, short neck, mild thoracic kyphoscoliosis	
Velinov (2008) [11]	12	M	-/-	G-banding, CGH, FISH	del(12)p13.3	6.2 Mb	ID, SCZ related psychosis	
Rooryck (2009) [12]	3	F	34/46	R-banding, CGH, qPCR	del(12)p13.33pter	2.3 Mb	DD, low birth weight, patent foramen ovale, short QT interval, microtia, preauricular tag and pit, wide left corner of the mouth, left macrosomia, oculoauriculovertebral spectrum	
MacDonald (2010) [13]	6	M	-/-	G-banding, MLPA, FISH	del(12)p13.3	2.95 Mb	ID, problems with social behavior, microcephaly, short nose, long face, prominent ears	
Abdelmoity (2011) [14]	8	F	-/-	G-banding, FISH, qPCR	del(12)p13.33interstital	1.39 Mb	DD, attention-deficit hyperactivity disorder	
Case 1 in Madrigal (2012) [15]	8	M	-/-	FISH, CGH	t(12;22)(p13.3;pter)	6 Mb	DD, global hypotonia, facial dysmorphism (rominent forehead, elongated face, bulbous nose with broad nasal root, small mouth and thin vermillion, mild hypertelorism and bilateral low-set ears with over-folded helices), ID, ADHD	
Case 2 in Madrigal (2012) [15]	27	F	-/-	FISH, CGH	t(12;22)(p13.3;pter)	6 Mb	DD, microcephaly, facial dysmorphism, ADHD	Maternal aunt of Case 1
Vargas (2012) [16]	12	F	25/35	G-banding, FISH, CGH	del(12)p13.33-p13.32	4.5 Mb	ID, psychotic disorder with hallucinations, arching eyebrow, malocculusion, gum hypertrophy, long and curving fingers, hypermobile joints	
Case 1 in Thevenon (2013) [17]	3	M	35/-	CGH, qPCR	del(12)p13.33-p13.32	3.2 Mb	DD, macrocephaly, coarse face, mild frontal bossing, enophalmia, low-set ear, marked philtrum, large nares, thin upper lip, irregular and narrowly spaced teeth	
Case 2 in Thevenon (2013) [17]	35	F	-/-	CGH, qPCR	del(12)p13.33-p13.32	3.2 Mb	Speech delay	Mother of Case 1
Case 3 in Thevenon (2013) [17]	5	M	-/37	G-banding, CGH, FISH	del(12)p13.33	1.3 Mb	DD, ID, behavioral abnormalities (hyperactivity, anxiety, solitariness, low social interaction)	
Case 4 in Thevenon (2013) [17]	37	M	-/67	CGH, FISH	del(12)p13.33	1.3 Mb	Speech delay	Father of Case 3
Case 5 in Thevenon (2013) [17]	67	M	-/-	CGH, FISH	del(12)p13.33	1.3 Mb	Speech delay, mild ID	Grandfather of Case 3
Case 6 in Thevenon (2013) [17]	10	M	-/-	CGH, FISH	del(12)p13.33	3.1 Mb	DD	Preterm birth, low birth weight
Case 7 in Thevenon (2013) [17]	11	M	-/-	CGH, FISH	del(12)p13.33	2.76 Mb	Long face with large ears and prominent lobes, epicanthus and large incisor with dental malocclusion, anxiety, and attention deficit hyperactive disorder	
Case 8 in Thevenon (2013) [17]	10	M	-/-	CGH	del(12)p13.33	2.5 Mb	ID, micrognathia, prominent ears, attention deficit hyperactivity disorder	Preterm birth, low birth weight
Case 9 in Thevenon (2013) [17]	16	M	-/-	CGH	del(12)p13.32-13.33interstital	4.76 Mb	ID, hypertelorism, microcephaly, joint laxity, brittle first toenails	
Fanizza (2014) [18]	5	M	26/29	G-banding, CGH, qPCR	del(12)p13.33-p13.32	2.3 Mb	Mild ID, speech delay, microcephaly, hypotonia, joint laxity	
Silva (2014) [19]	8	M	30/30	G-banding, CGH, FISH	del(12)p13.33	1.5 Mb	Spina bifida occulta, ASD	
Faria (2016) [20]	9	F	-/-	G-banding, CMA, FISH	del(12)p13.33-13.32	4.2 Mb	ID, borderline microcephaly, small face, small downslanting palpebral fissures, protruding ears and overfolded ear helices, high nasal root, overbite, high narrow palate, retrognathia, irregular dental implantation, clinodactyly on the 5th finger	
Leyser (2016) [21]	4	M	-/-	G-banding	del(12)p13.2	-	DD, ASD	Alcohol consumption
ID_358 in Quintela (2017) [22]	1	M	-/-	CMA	del(12)p12.2-11.23	6.27 Mb	DD, ID, facial dysmorphism, perinatal ponderal delay	
ID_45 in Quintela (2017) [22]	15	M	-/-	CMA	del(12)p13.33	204.5 Kb	Mild ID, ADHD, behavior problems	
Mio (2020) [23]	7	M	-/-	CGH, qPCR	del(12)p13.33	44.7 Kb	Expressive language impairment, tremors, fine motor-skills delay, muscular hypotonia, and joint laxity	
Our proband	12	F	40/42	NGS, CMA	del(12)p13.33-13.32	3.2 Mb	ID, epilepsy	
Our proband’s mother	40	F	-/-	NGS, CMA	del(12)p13.33-13.32	3.2 Mb	ID, SCZ	

yr in Age, year; M in Sex, Male; F in Sex, Female; M in Parental age, mother; F in Parental age, father; -, not available; Q-banding, Quinacrine banding; G-banding, Giemsa banding; R-banding, reverse banding; CGH, comparative genomic hybridization; FISH, fluorescence in situ hybridization; qPCR, quantitative real-time PCR; MLPA, multiplex ligation-dependent probe amplification; NGS, next-generation sequencing; CMA, chromosomal microarray; ID, intellectual disability; DD, developmental delay; SCZ, schizophrenia; ASD, autism spectrum disorder.

## Data Availability

The data presented in this study are available on request from the corresponding author.
